# Supplementary material for: Association between oral microbiome and seven types of cancers in East Asian population: a two-sample Mendelian randomization analysis
Source: Front Mol Biosci. 2023 Nov 21;10:1327893. doi: 10.3389/fmolb.2023.1327893 (PMC10702768; doi:10.3389/fmolb.2023.1327893)
Supplement: Supplementary file 1 [file DataSheet1.ZIP › Supplementary Materials/MR plots for tongue/tongue═╝/Ovarian cancer/pheno.1423_to_ovarian cancer_forest.pdf]

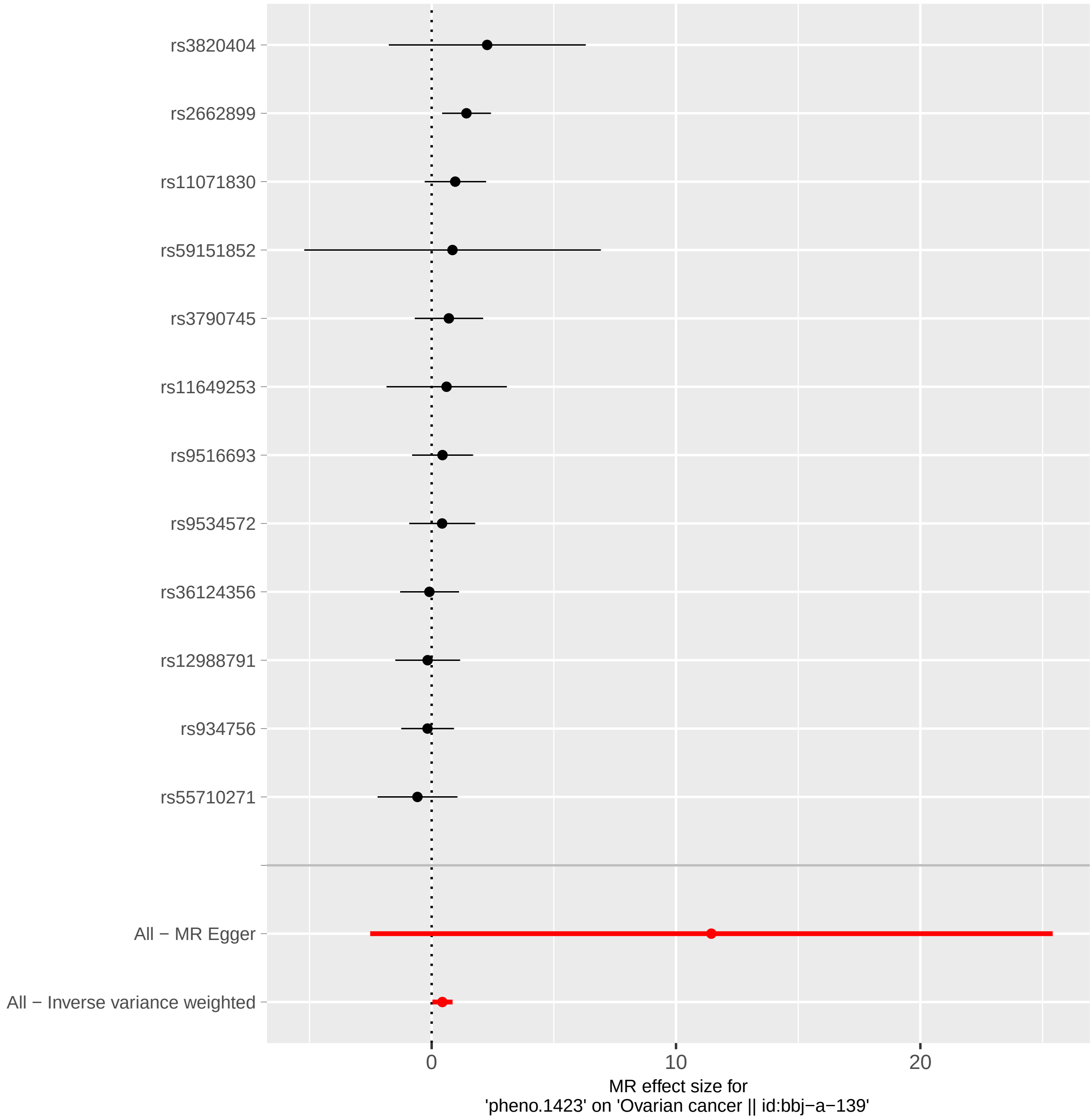
rs3820404
rs2662899
rs11071830
rs59151852
rs3790745
rs11649253
rs9516693
rs9534572
rs36124356
rs12988791
rs934756
rs55710271
All − MR Egger
All − Inverse variance weighted
0
10
20
MR effect size for
'pheno.1423' on 'Ovarian cancer || id:bbj−a−139'
